# PVsiRNAdb: a database for plant exclusive virus-derived small interfering RNAs

**DOI:** 10.1093/database/bay105

**Published:** 2018-10-17

**Authors:** Nikita Gupta, Shafaque Zahra, Ajeet Singh, Shailesh Kumar

**Affiliations:** Bioinformatics Laboratory, National Institute of Plant Genome Research, Aruna Asaf Ali Marg, New Delhi, India

## Abstract

Ribonucleic acids (RNA) interference mechanism has been proved to be an important regulator of both transcriptional and post-transcription controls of gene expression during biotic and abiotic stresses in plants. Virus-derived small interfering RNAs (vsiRNAs) are established components of the RNA silencing mechanism for incurring anti-viral resistance in plants. Some databases like siRNAdb, HIVsirDB and VIRsiRNAdb are available online pertaining to siRNAs as well as vsiRNAs generated during viral infection in humans; however, currently there is a lack of repository for plant exclusive vsiRNAs. We have developed `PVsiRNAdb (http://www.nipgr.res.in/PVsiRNAdb)’, a manually curated plant-exclusive database harboring information related to vsiRNAs found in different virus-infected plants collected by exhaustive data mining of published literature so far. This database contains a total of 322 214 entries and 282 549 unique sequences of vsiRNAs. In PVsiRNAdb, detailed and comprehensive information is available for each vsiRNA sequence. Apart from the core information consisting of plant, tissue, virus name and vsiRNA sequence, additional information of each vsiRNAs (map position, length, coordinates, strand information and predicted structure) may be of high utility to the user. Different types of search and browse modules with three different tools namely BLAST, Smith–Waterman Align and Mapping are provided at PVsiRNAdb. Thus, this database being one of its kind will surely be of much use to molecular biologists for exploring the complex viral genetics and genomics, viral–host interactions and beneficial to the scientific community and can prove to be very advantageous in the field of agriculture for producing viral resistance transgenic crops.

## Introduction

The emergence of high-throughput, fast and cost-effective next generation sequencing (NGS) technology has facilitated the study of small non-coding ribonucleic acids (RNAs) in eukaryotes and their role in RNA silencing mechanisms as a defense response during pathogen infection. Among different causal agents of infection, virus-mediated infections have a significant impact on the physiology, nutritional value and productivity of crop plants (1, 2). Thus, it becomes important to focus on the underlying anti-viral defense mechanisms. Some important natural anti-viral defenses exploit small RNAs in combating the infection in host plants (3), preferably termed as virus-induced gene silencing (VIGS). VIGS also assists in the process of chromatin modification, translation process and thus a potent mediator for gene expression regulation bestowing the overall resistance in host plants against the viral defense. They have gained considerable attention in recent years by plant researchers and small interfering RNAs (siRNAs) being an integral component of VIGS have been extensively studied in plants (4, 5). In diverse eukaryotes including plants, virus infection induces the generation of small RNA molecules, which may lead to either develop anti-viral immunity or occurrence of disease in some cases (6). A majority of these RNA molecules comprises of siRNAs with length ranging from 21 to 24 nucleotide (nt) with 3′-unphosphorylated overhangs of 2 nt are known to be the potential regulators of gene expression as a part of a natural anti-defense system for the host plant (7).

It has been elucidated from previous research studies that these virus-derived siRNAs (vsiRNAs) in plants can be derived from both RNA and deoxyribonucleic acid (DNA) viruses (8). Further, vsiRNAs can be derived from single-stranded (ss) RNA with hairpin-shaped folded structure, double-stranded (ds) RNA with the preference for the sense strand of genomic RNA or from dsDNA intermediates (for DNA viruses), formed during replication stage inside the host cell (9, 10). The Dicer-like enzymes (DCLs) are involved in the processing of viral genomes for the biogenesis of siRNAs in plants (11, 12). DCL homologs namely DCL-2, 3 and 4 are directly involved in the production of vsiRNAs whereas DCL-1 acts in an indirect manner for biogenesis of different vsiRNAs (4). These DCLs have distinct functionality for vsiRNAs production and they perform their functions in a coordinated and self-balanced way. The DCLs that act directly on viral genomes give rise to primary vsiRNAs. These primary vsiRNAs associate themselves with Argonaute (AGO) proteins forming an RNA-induced silencing complex (RISC) (13). RISC targets viral genome with the aid of plant RNA-dependent RNA polymerases (RDRs). The viral genome gets fragmented in small chunks of dsRNA (initial phase) mediated by DCLs and further action of RDRs lead to the production of functionally active secondary vsiRNAs during the amplification phase (14).

**Figure 1:**
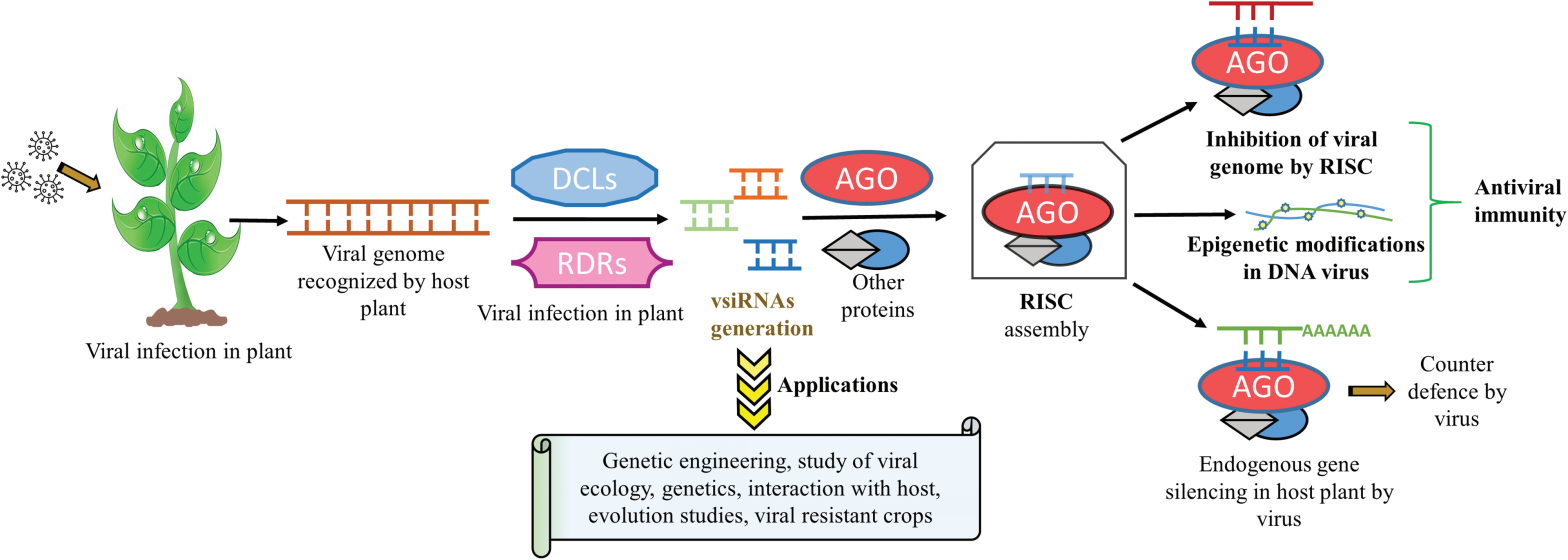
Biogenesis, mode of action of plant vsiRNAs, their applications and scope in biotechnology.

**Figure 2:**
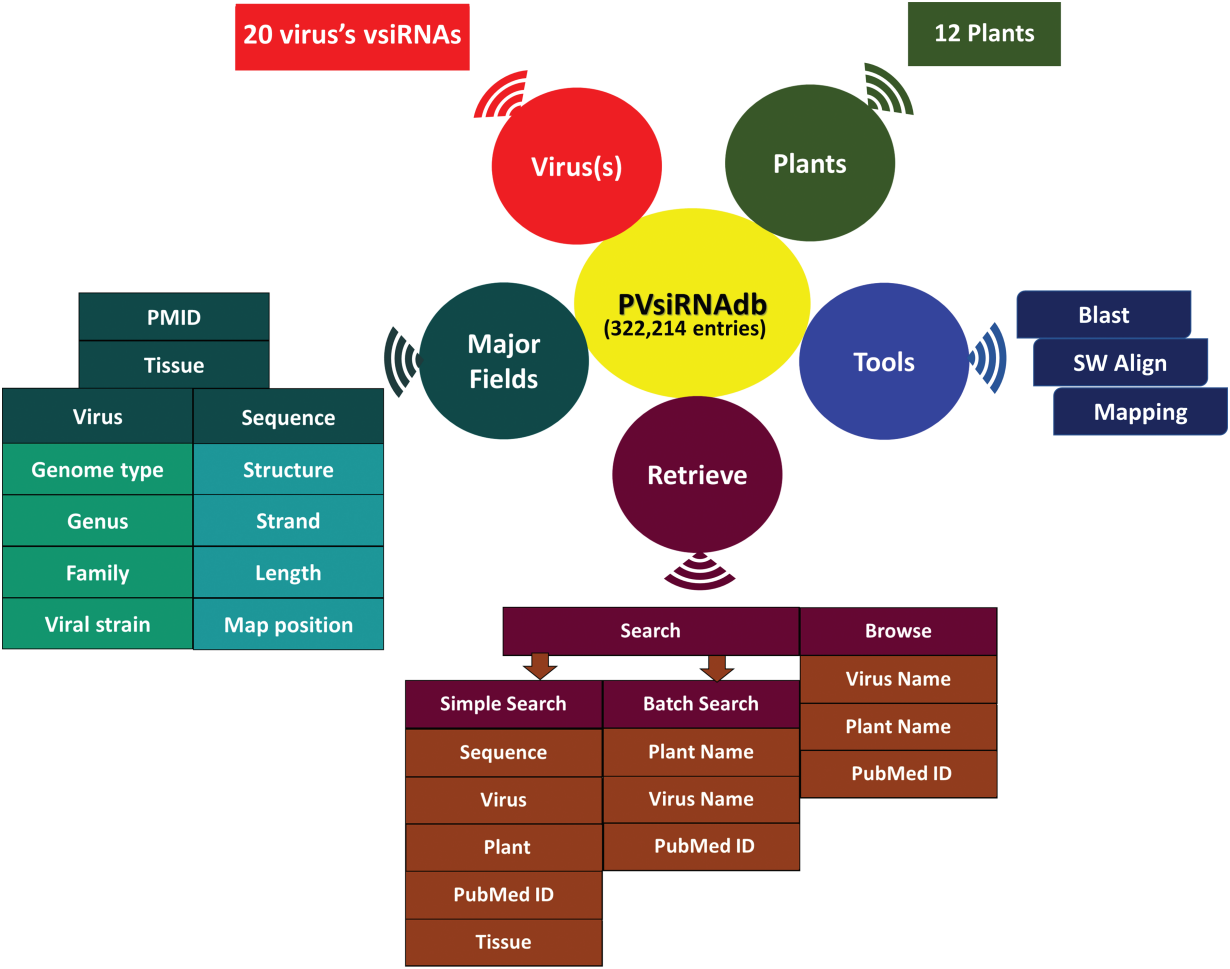
Overall representation of PVsiRNAdb.

In plants, vsiRNAs after assembling with RISC act in a sequence-specific manner and bind to its homologous complementary viral genomic RNA or genomic DNA transcript and thereby silencing viral expression and imparting anti-viral resistance to the host plant (8, 15). Although less explored, they are also supposed to regulate cellular processes via epigenetic modifications like DNA methylation of gene promoters (16). Apart from their pivotal role in transcriptional gene silencing (TGS) and post-TGS (PTGS), siRNAs can also be artificially synthesized and exploited for gene knockout and studies related to various pathways associated with gene silencing under various types of stress conditions (7). The development of deep sequencing technology has been utilized for studying viral genomes, ecological studies, genetics, viral–host interaction and co-evolution of viruses with their hosts using vsiRNAs (17, 18). For example, by using the hairpin structure construct approach, vsiRNAs can be artificially expressed in plants and can be exploited specifically for targeting against the particular pathogen (19, 20). Transgenic plants resistant to multiple viruses have also been developed using multiple hairpin RNA constructs generated from different viral sources (21, 22). This has paved the way of giving new dimensions to RNA interference (RNAi) technology for developing viral-resistant plants, which is applicable for both agriculture and horticulture (23). The summary of the generation of vsiRNAs and some of their important applications are illustrated in Figure 1.

The availability of a knowledge base dedicated to the plant vsiRNAs is anticipated to assist the scientific community for better understanding of vsiRNA generation and their action and further designing of more effective silencing strategies for developing viral resistant plants. Currently, a number of databases like siRNAdb (24), HIVsirDB (25) and VIRsiRNAdb (26) are available online pertaining to siRNAs as well as vsiRNAs detected in several lower to higher organisms, with emphasis on viral diseases related to humans. Considering the wide impact of vsiRNAs on the gene regulation in plants, there is still a lack of knowledge base related to plant vsiRNAs. Thereby, there is a need to develop an exclusive repository for vsiRNAs detected in plants. In this study, we have developed a database of plant-exclusive virus-derived siRNAs named PVsiRNAdb. PVsiRNAdb (http://www.nipgr.res.in/PVsiRNAdb) is developed by extensive data mining and harboring information of plant vsiRNAs from literature available till date. We have extracted the resources available online regarding vsiRNAs (considering both predicted as well as annotated sequences) generated in plants by different virus infections and performed sequence- and structure-related analyses using a bioinformatics approach. PVsiRNAdb is developed in such a manner so that it is convenient to access with user-friendly options. The overall representation of PVsiRNAdb is shown in Figure 2.

## Materials and methods

### Data procurement

A comprehensive literature search was carried out to extract the relevant articles from PubMed (https://www.ncbi.nlm.nih.gov/pubmed). This was carried out by searching queries having a combination of different keywords e.g. viral siRNAs, plant–virus interaction, siRNA, plant-viral siRNAs etc. All the articles were manually screened for relevant experimental information. Further reviews and articles lacking relevant information were excluded. Full-text search was done for each of the relevant article having the information of plant-specific vsiRNAs. Further, information regarding the plant, tissue, PubMed ID (PMID) and PVsiRNA-ID was incorporated with the collected information of vsiRNAs.

### PVsiRNAdb

The web interface of PVsiRNAdb was built on an Apache Hypertext Transfer Protocol server by using Hypertext Markup Language, Cascading Style Sheets, Hypertext Preprocessor (PHP) and JavaScript. MySQL, an object-relational database management system (RDBMS), was used to manage all the data in the backend. It provides commands to retrieve and store the data in the database. All common gateway interface and database interfacing scripts were written in the Hypertext Preprocessor (PHP); and Practical Extraction and Reporting Language (PERL).

### Database organization

`PvsiRNAdb’ holds information related to vsiRNAs at two levels, namely primary and secondary. At the primary level, the user can search their queries by using simple keywords such as the name of a particular virus strain or plant name as per the requirement. The information will be displayed according to the number of fields selected by the user. The user can also search multiple queries for virus, plant or PMIDs by performing a batch search. The data at the secondary level can be utilized for the retrieval of further information about primary data. At the secondary level, additional information like experimental details, sequence information and viral details like name, genome type as well as classification can also be fetched for each viral strain. The virus name, genome type as well as classification can also be fetched for each viral strain. The specific details about any experiment can be inquired by clicking on PMID hyperlink, which will direct the user to original link of that research article. As structure plays an important role in determining the function of any sequence, the secondary structure of vsiRNA sequences was added to the database using in-house generated PERL scripts for running the Mfold (31) and RNAstructure (32) software packages. Mfold was used for calculating minimized energy for the folded structure and structure coordinates were predicted by Draw utility of RNAstructure software.

### PVsiRNAdb features and data retrieval tools

In PVsiRNAdb, detailed and comprehensive information is incorporated for each siRNA entry. Apart from the core information including the siRNA sequence, siRNA length, virus name and plant name, additional information like PMID, plant tissue, mapping coordinates of siRNA to the plant genome and the predicted secondary structures of siRNA may be of high utility to the user.

PVsiRNAdb provides two user-friendly options to search for siRNA information. First option `Simple Search’ facilitates the user to query the siRNA by providing different search terms including the name of the virus, plant name (scientific or common name), siRNA sequence, PMID and PVsiRNA-ID. To provide the flexibility, two options i.e. `containing’ and `exact’ have been incorporated for search terms. This option also facilitates the user to select the fields to be displayed. A total of five display fields are available for a particular search term. Three display fields namely the `Virus name’, `PMID’ and 'Sequence' are further linked with their corresponding information. Second option to search in PVsiRNAdb is that of the `Batch Search’ providing the facility to search for multiple queries at a time. The user can extract the information of siRNAs by providing a list of plant names, virus names or PMIDs. In this module, an example list of all the three search terms is provided for the users. The `Browse’ section of PVsiRNAdb facilitates the user to extract information by three different approaches (browse by virus name or plant type or by PMID).

In the `Tools’ section, we have incorporated three different modules for the analysis of sequences provided by the user e.g. `BLAST’, `SW Align’ and `Mapping’. A query sequence can be aligned to the siRNA sequences of PVsiRNAdb using the `BLAST’ module incorporated in this database. This section has the facility to select the virus name to which the user wishes to align the input sequences. Query sequences are aligned to the vsiRNAs of the selected virus. Different options of `E value’ are also available in this section for BLAST search to know the significance of the match. The `SW Align’ module facilitates the user to perform optimal local alignment of the query sequence either to complete viral genome or onto the different sets of siRNAs derived from different viruses. For this purpose, the Smith–Waterman algorithm has been integrated into this module and output results displayed with the help of in-house developed PERL scripts. The `Mapping’ module is designed for the mapping of siRNA sequences, available at PVsiRNAdb to the user-provided sequences e.g. messenger RNA sequences or genomic sequences. This facility is useful for the designing of specific siRNAs corresponding to the specific viral genome.

The `STATISTICS’ section displays the overall enumeration of siRNAs incorporated at PVsiRNAdb. Another important section of PVsiRNAdb web interface is `Help/Guide’. This section has different subsections namely `Help’, `Links’ and `References’. From the `Help’ subsection of PVsiRNAdb, the user can understand the working of this database with the help of self-explanatory figures. `Links’ harboring information of important web resources for viral siRNAs. In the `References’, we have incorporated all the articles related to vsiRNAs involved in plant–viral interactions.

## Result and discussions

**Table 1:** List of virus, host plant, total and unique vsiRNAs stored in PVsiRNAdb

**Virus**	**Host plant**	**Total vsiRNAs**	**Unique vsiRNA sequences**
Bamboo mosaic virus	*Dendrocalamus latiflorus* (bamboo)	18	18
Brassica yellow virus	*Nicotiana benthamiana* (tobacco)	143	100
Chinese wheat mosaic virus	*Triticum aestivum* (wheat)	19 536	18 585
Cotton leaf curl Multan virus	*Gossypium hirsutum* (cotton)	4736	4736
Cucumber green mottle mosaic virus	*Cucumis sativus* (cucumber)	92	88
Cucumber mosaic virus	*Arabidopsis thaliana* (thale cress)	47	47
Cymbidium ringspot virus	*Nicotiana benthamiana* (tobacco)	12 305	5938
Grapevine fleck virus and Grapevine rupestris stem pitting associated virus	*Vitis vinifera* (grapevine)	62	62
Maizechlorotic mottle virus and Sugarcane mosaic virus	*Zea mays* (maize)	260	200
Pea enation mosaic virus 2	*Nicotiana benthamiana* (tobacco)	137	100
Potato virus Y	*Solanum tuberosum* (potato)	46 435	46 435
Prunus necrotic ring spot virus	*Prunus avium* (cherry)	152	134
Rice black-streaked dwarf virus	*Oryza sativa* (rice)	468	172
Southern Rice black streaked dwarf virus	*Oryza sativa* (rice)	20 876	20 606
Sugarcane mosaic virus	*Zea mays* (maize)	100	100
Tobacco mosaic virus	*Nicotiana benthamiana* (tobacco)	682	445
Tobacco rattle virus	*Nicotiana benthamiana* (tobacco)	142	140
Wheat yellow mosaic virus	*Triticum aestivum* (wheat)	216 018	184 699
Zucchini yellow mosaic virus	*Citrullus lanatus* (watermelon)	5	5

**Figure 3:**
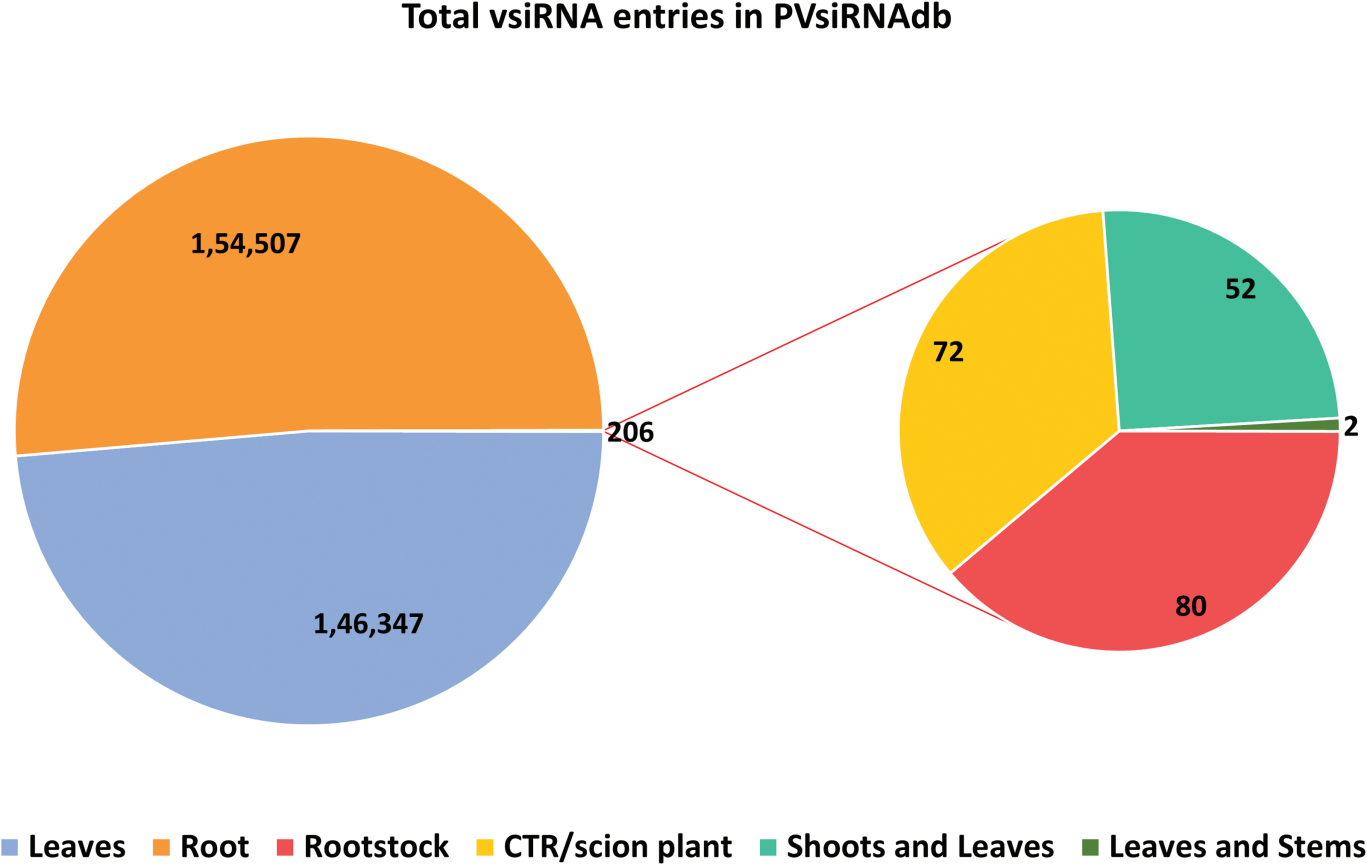
Tissue-wise distribution of total vsiRNA sequences.

**Figure 4:**
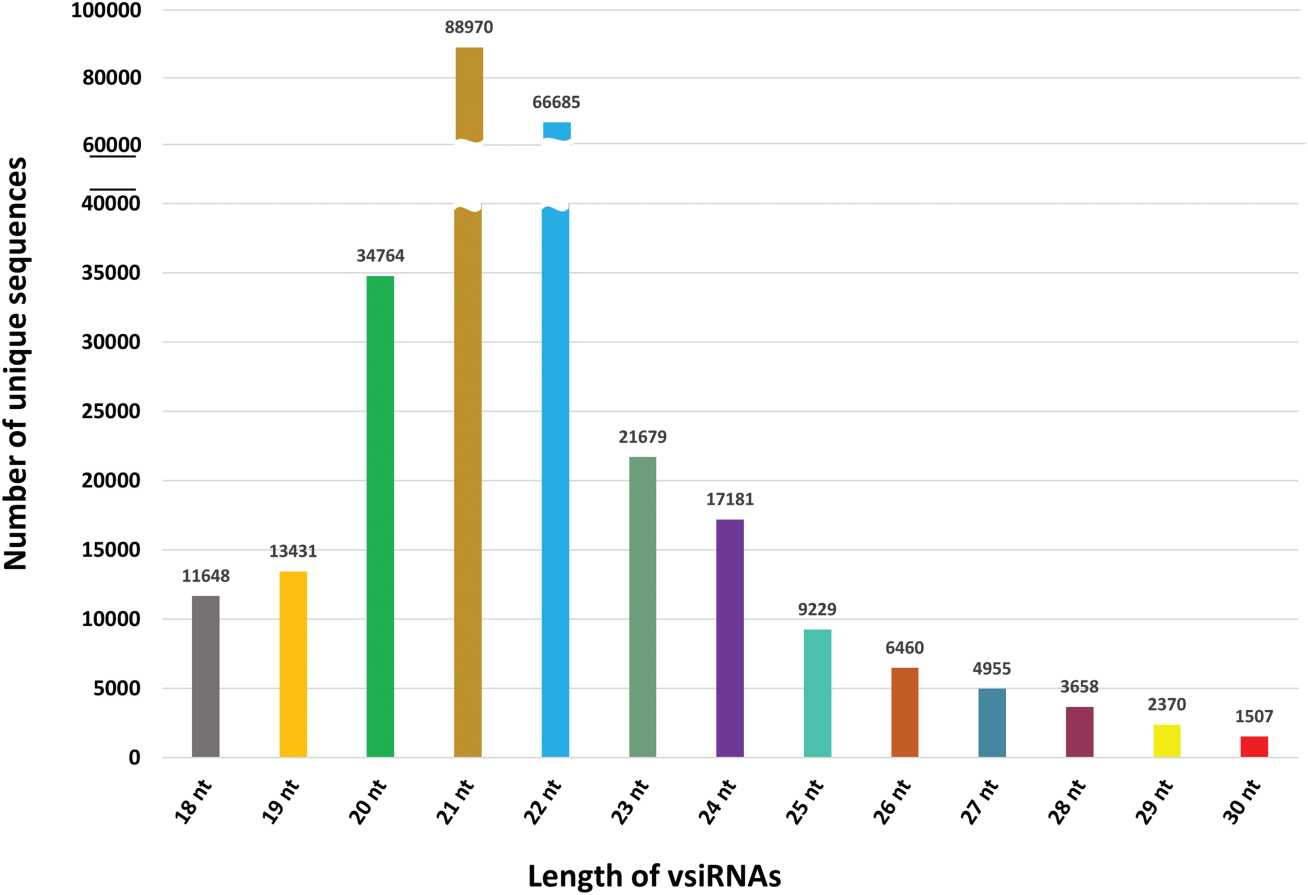
Length-wise distribution of all unique vsiRNA sequences in PVsiRNAdb.

PVsiRNAdb holds detailed information about vsiRNAs with a total of 322 214 entries and 282 549 unique vsiRNAs sequences derived from 20 different viral strains infecting 12 different plant species as illustrated in Table 1. For better understanding and analysis, the vsiRNAs have been classified under different categories like the virus-wise classification of total and unique vsiRNA sequences (Table 1), tissue-wise distribution (Figure 3) and length-wise differentiation (Figure 4) of unique vsiRNA sequences. The vsiRNAs extracted from roots and leaves outnumbered those vsiRNAs that were obtained from other tissues (Figure 3). This reflects the possibility of the existence of tissue-specific enzymes for the biogenesis of vsiRNAs. It was observed that most vsiRNAs fall in the length ranging from 19 to 24 nt supporting other previous literature (27, 28). The tissue-wise distribution of vsiRNAs, as well as length-wise categorization of these vsiRNAs, has been graphically illustrated in the statistics section of the database. Furthermore, the detailed information pertaining to each vsiRNA sequence along with secondary structure has also been incorporated into the PVsiRNAdb.

In recent times, plant vsiRNAs have captured remarkable attention of molecular biologists as they have been proved to be potential agents for incurring anti-viral immunity in plants and, interestingly, they are also shown to be associated with viral suppressors of RNA silencing as a strategy of endogenous gene silencing for counter defense by virus (29). NGS technology has paved a way for better understanding the complex world of vsiRNAs. This database is believed to be highly resourceful for the molecular biologists dedicated to the broad field of RNAi study and will be of great value for exploring the role and pathways of major proteins like AGO and RDRs in gene silencing pathways and in elucidating the cross-talks among different silencing pathways. Apart from silencing strategy of plants for host defense responses, interestingly, vsiRNAs also play role in counter defense mechanism by the viruses by targeting specific host genes, production of suppressor proteins (15) and specific sequences like beta-satellite sequences against the host plant (30). This database will ease in the study of viral strategies for adapting inside the host plants and in designing the artificial siRNA for gene silencing and combinatorial approaches using different silencing pathways like transcriptional gene silencing (TGS), microRNAs, Piwi-interacting RNAs (piRNAs) etc. for designing more effective silencing strategies for viral resistant plants. This will also ease the understanding of the common conserved mechanism of RNA-mediated PTGS found in eukaryotes and targeting of desired host genes using vsiRNA machinery induced by viruses.

Thus, this database holds a repository of currently available information related to plant vsiRNAs and, with the scope of further addition of more entries, is believed to be beneficial for virologists and plant breeders for improving the crop plants with better resistance against single or multiple viruses. However, this is just the starting point in the vsiRNAs research as a detailed mechanism of their mode of action and downstream processes are yet to be explored in the plant kingdom. As the plant–virus interaction is a complex area of research, the in-depth study of vsiRNAs can prove to be advantageous in unraveling the viral genomes and genetics, the mechanism of RNAi as well as repercussions of virus–host interaction.

## Author contributions

N.G. and S.K. developed the web interface of the database. N.G., S.Z. and A.S. collected and compiled the data and performed the analysis. S.Z. and S.K. wrote the manuscript. S.K. conceived the idea and coordinated the project.

## Notes

**Database URL:** http://www.nipgr.res.in/PVsiRNAdb
